# A proteomic-based approach to study underlying molecular responses of the small intestine of Wistar rats to genetically modified corn (MON810)

**DOI:** 10.1007/s11248-019-00157-y

**Published:** 2019-06-06

**Authors:** Asmaa AL-Harbi, Sahira Lary, Martin G. Edwards, Safaa Qusti, Andrew Cockburn, Morten Poulsen, Angharad M. R. Gatehouse

**Affiliations:** 1 Biochemistry Department, Faculty of Science, King Abdulaziz University, P.O. Box: 42805, Jeddah, 21551 Kingdom of Saudi Arabia; 2 School of Natural and Environmental Sciences, University of Newcastle, Newcastle upon Tyne, NE1 7RU UK; 3 The National Food Institute, Technical University of Denmark, 2800 Kgs. Lyngby, Denmark

**Keywords:** MON810, *Bacillus thuringiensis*, Cry1Ab protein, Wistar rats, Feeding trials, Epithelial cells, Proteome, Stress-proteins

## Abstract

A genetically modified (GM) commercial corn variety, MON810, resistant to European corn borer, has been shown to be non-toxic to mammals in a number of rodent feeding studies carried out in accordance with OECD Guidelines. Insect resistance results from expression of the Cry1Ab gene encoding an insecticidal *Bt* protein that causes lysis and cell death in susceptible insect larvae by binding to midgut epithelial cells, which is a key determinant of Cry toxin species specificity. Whilst whole animal studies are still recognised as the ‘gold standard’ for safety assessment, they only provide indirect evidence for changes at the cellular/organ/tissue level. In contrast, omics-based technologies enable mechanistic understanding of toxicological or nutritional events at the cellular/receptor level. To address this important knowledge-gap and to gain insights into the underlying molecular responses in rat to MON810, differential gene expression in the epithelial cells of the small intestine of rats fed formulated diets containing MON810, its near isogenic line, two conventional corn varieties, and a commercial (Purina™) corn-based control diet were investigated using comparative proteomic profiling. Pairwise and five-way comparisons showed that the majority of proteins that were differentially expressed in the small intestine epithelial cells in response to consumption of the different diets in both 7-day and 28-day studies were related to lipid and carbohydrate metabolism and protein biosynthesis. Irrespective of the diet, a limited number of stress-related proteins were shown to be differentially expressed. However these stress-related proteins differed between diets. No adverse clinical or behavioural effects, or biomarkers of adverse health, were observed in rats fed GM corn compared to the other corn diets. These findings suggest that MON810 has negligible effects on the small intestine of rats at the cellular level compared with the well-documented toxicity observed in susceptible insects.

**Electronic supplementary material:**

The online version of this article (10.1007/s11248-019-00157-y) contains supplementary material, which is available to authorized users.

## Introduction

Since their commercialization in the mid 1990s, there has been global interest surrounding the use of Biotech crops (Genetically Modified (GM), transgenic crops) and their contribution to sustainable food production (James 2015). Assurance of their safety for human and animal consumption is key to their acceptability and there remains widespread and vocal demand for additional evidence of their safety (Haslberger 2006; Hug 2008). Numerous trials with animals fed different diet formulations based on GM crops such as corn, rice, soybeans, potatoes, and tomatoes have been conducted, and parameters such as food consumption, body weight, organ weight, blood chemistry, and histopathology have been measured. Many recommendations have been made as to robust methods required for safety testing (Hammond 2007; Koch et al. 2015). There is a growing consensus that safety assessment and toxicology are changing, and thus new strategies for evaluating risk that are less dependent upon apical toxicity endpoints in animal models, but rely more on knowledge of the mechanism of toxicity, are required (Daneshian et al. 2013; Blaauboer et al. 2016).

Insect resistance represents a major trait expressed in transgenic crops, either when expressed singly or combined with other traits such as herbicide tolerance (James, 2016). Since the production of corn, which is both an important food and feed crop, is severely constrained by insect attack, it was an ideal candidate for genetically engineering for insect resistance. Transgenic corn (Event MON810), expressing the *Bacillus thuringiensis* Cry1Ab protein, was shown to be highly resistant to the European corn borer and licensed widely to the seed industry under the brand name YieldGard (Coram et al. 2016). This event was also shown to be tolerant to fungal pathogens including *Fusarium* sp. as a consequence of reduced insect damage (Nedelnik et al. 2012). The mode of action of Cry proteins has been extensively studied and different models put forward. Cry proteins show a high level of target species-specific toxicity, with only a few insect species being affected by each of the different Cry proteins (Crickmore et al. 2015). Cry1Ab is a member of the 3-d class of pore-forming toxins that cause cell death by forming ionic pores in the membrane of the midgut epithelial cells of their target insect (Betz et al. 2000). Although competing models differ in post-binding events that eventually kill insects, the currently accepted paradigm asserts that protoxins must be proteolytically cleaved to form a truncated active toxin, which then binds to the high affinity receptor cadherin. The interaction of the monomeric Cryl A toxin with the cadherin receptor promotes further proteolytic cleavage, where helix alpha-1 of domain I is removed resulting in toxin oligomerization. The oligomeric structure then binds to a secondary receptor, aminopeptidase N or alkaline phosphatase, enabling the toxin to become inserted into the membrane resulting in osmotic shock, cell lysis and subsequent death of the insect (Soberón et al. 2010). However, recent studies suggest that protoxins can be more toxic to certain insects leading the authors to propose a dual model in which protoxins and activated toxins kill insects via different pathways (Tabashnik et al. 2015). In contrast, Cry proteins are regarded as nontoxic to mammals, including humans, possibly due to acidified gut pepsinolysis and the lack of specific high-affinity Cry protein receptors on the GI-tract epithelial surface of mammals, including humans (Vachon et al. 2012); this may be due, in part, to the absence of BL2, a glycosylating enzyme present in the gut cells of invertebrates responsible for the production of specific sugar residues that facilitate recognition and binding by Cry proteins. Numerous mammalian toxicity studies show no significant adverse effects of the Cry proteins on body weight gain or clinical observations (McClintock et al. 1995). The safety of MON810 has been evaluated in previous studies that reported no toxicologically significant differences in clinical and neurobehavioural signs, ophthalmology, clinical pathology, organ weights, and gross and microscopic pathology between rats fed the transgenic and conventional corn (Hammond et al. 2006; MacKenzie et al. 2007). Furthermore, allergenicity studies for the Cry1Ab protein have been carried out in humans; sensitive subjects reacted no differently to GM and non-GM samples either by skin prick tests or in terms of IgE immunoreactivity (Finamore et al. 2008). However, despite the large body of evidence pointing to the absence of histopathological abnormalities in tissues of animals fed GM-based corn diets, or indeed clinical effects (Hammond et al. 2006; MacKenzie et al. 2007; Bartholomaeus et al. 2013; Domingo 2016) there remains considerable concern by stakeholder groups, including risk assessment bodies, as to the safety of such products (Antoniou and Robinson 2017). What is surprising is the lack of evidence of any studies to investigate the potential effects of *Bt* present in corn-based diets at the subcellular or molecular level, and in particular, in comparable target tissues in mammals to those present in susceptible insects where the Cry proteins are known to bind (Pigott and Ellar 2007).

Whilst whole animal studies are still recognised as the ‘gold standard’ for safety assessment, such studies have the limitation of only obtaining indirect evidence for changes at the cellular, organ or tissue level. In contrast, omics-based technologies enable detailed studies at the cellular/receptor level, and so have the potential to permit mechanistic understanding of toxicological or nutritional events. To address this important knowledge-gap and to gain insights into the underlying molecular responses in rat to MON810, differential gene expression in the epithelial cells of the small intestine (S.I.) of rats fed formulated diets containing MON810 were investigated using comparative proteomic profiling. As comparators we used the near isogenic parental line, two conventional corn varieties, and a corn-based control diet. These studies also allowed us to search for biomarkers of exposure or effect using pairwise and five-way analyses. We report here the results of toxicological analyses of rats fed different corn-based diets, including the transgenic *Bt* line MON810 in both 7-day and 28-day studies. We also report the findings of the proteomics studies on the S.I. epithelial cells of rats fed those diets, with a focus on stress-related proteins and discuss the biological relevance of the results obtained. In these studies rats were used as an experimental model for both humans and livestock.

## Materials and methods

### Test materials

Transgenic *Bt* corn MON810, its corresponding parental non-transgenic (near isogenic) corn (MON Conv Corn), and two different corn varieties, MON Garst 8450 as reference 1, and MON Gold HVST H8920 as reference 2 were kindly provided by Monsanto, St Louis, USA and commercial Purina rodent chow (MCert Rod control), by Purina Mills. All diets were formulated by TestDiet and prepared by Purina. Approximately 33% (w/w) corn grain were used for all diets, adjusting other components of the diet to provide approximately equal levels of protein, calories and nutrients in all diets (See Table S1 for diet composition); this ensured a nutritionally balanced rodent diet for both the 7-day and 28-day feeding studies. Allocation of rats to different experimental groups is shown in Table S2. Compositional, contaminant, and nutritional content of the experimental diets conformed to specifications for Certified Rodent LabDiet 5002 established by Purina Mills International (PMI). PCR analysis confirmed that the test diet contained MON810 as it tested positive for the Cry1Ab transformation event. The control and reference diets did not test positive for the Cry1Ab transformation event and therefore were considered to be free from contamination by the transgenic line.

### Animals and housing

Prior to the start of the study, 40 young adult male Wistar rats, between 6 and 7 weeks, were allowed to acclimate to the housing conditions for 1 week during which they were fed basal diet as previously described. Rats were housed individually in polycarbonate cages and maintained 12 h light/dark cycle (the photoperiod began from 07.00 a.m.), at a temperature of 23 ± 3 °C and a relative humidity of 47% ± 5; food and tap water were provided ad libitum. Animals were observed twice daily for general wellbeing. These studies conformed to the National Research Council guidelines for animal experimentation (National Research Council. Guide for the care and use of laboratory animals 2010).

### Experimental design: 7-day and 28-day rat feeding trials

Two animal feeding studies were conducted under the conditions described above. The study design was based on OECD guideline No. 407 (OECD 2008) for a repeated dose 28-day oral toxicity study in rodents. Rats were randomly assigned to five experimental groups based on body weight means and fed with the different experimental diets for either 7 or 28 days. Four rats per group were used for both 7- and 28-day feeding trials (Table S2). Water was freely available throughout the study. The body weight and cumulative food consumption for each individual was recorded daily and regular observations were made regarding animal development and behaviour. Visual assessment was used to monitor behavioural changes in accordance with the National Research Council Guidelines (https://www.nap.edu/catalog/1542/recognition-and-alleviation-of-pain-and-distress-in-laboratory-animals). Body weights were analyzed by ANOVA, followed by Tukey Test.

### Isolation of small intestine epithelial cells

Intestinal epithelial cells (IECs) were isolated from the rat small intestine (SI) according to Bjerknes and Cheng (1981) with minor modifications. At the end of each trial the animals were killed by cervical dislocation. The abdomen of each rat was opened, the SI removed and the lumen perfused with cold phosphate-buffered saline (PBS), pH 7.4 and gently everted using a 4-mm-diameter glass rod and flushed with PBS with its two ends enveloped. The intestine was transferred to a clean flask, immersed into warm PBSPE solution (PBS containing 1 mM PMSF and 1 mM EDTA), shaken at 150 cycles per min for 5 min, transferred to another clean flask containing warm PBSPE solution, and again agitated for 5 min. The shed IECs were centrifuged at 500×*g* for 10 min. The cell pellets were harvested and finally washed in ice-cold PBS three times. Cell counts of the isolated viable epithelial cells were performed using a cell analyser (BECKMAN Coulter Counter, U.S.A) and ViCellXR 2.03 software.

### Proteomics

#### Protein extraction from isolated rat intestinal epithelial cells

Total proteins were extracted from the isolated IECs with 200 µl of lysis buffer containing 7 M urea, 2 M thiourea, 65 mM DTT, 2% CHAPS, 2 mM PMSF, 0.5% IPG buffer, and protease inhibitor mixture (GE healthcare). The extraction mixture was sonicated using Sonic Dismembrator (Fisher Scientific), and then centrifuged at 12,000×*g* at 4 °C for 20 min. Protein quantification and clean-up were carried out according to the manufacturer’s instructions (GE Healthcare, Sweden). Isoelectric focusing (IEF) and SDS-PAGE were carried out according to the manufacturer’s instructions on an Ettan IPGphor 3 isoelectric focusing unit (GE Healthcare, Sweden) and PROTEAN^®^ II xi Cell (Bio-Rad, U.S.A) with a chiller circulator (JULABO, U.S.A). IEF was conducted at 20 °C on 18 cm IPG strips with a linear gradient (pH 3–10), as previously described (Ferry et al. 2011). Initially, the samples were run in step mode for 2 h at 250 V followed by gradient mode of 10,000 V for 3 h, and finally, step mode of 10,000 V for 4 h. The focussed IPG strips were equilibrated for 15 min in 10 mL equilibration solution (50 mM Tris-HCl, 6 M urea, 30% w/v glycerol, 2% w/v SDS, 0.002% w/v bromophenol blue, and 1% w/v DTT) followed by equilibration in buffer containing 2.5% w/v iodoacetamide for another 15 min. Strips were transferred to a vertical SDS–polyacrylamide gel and the second dimension performed on a 12.5% SDS–polyacrylamide gels at 4 °C; proteins were stained with colloidal Coomassie blue G-250 (Sigma).

#### Image and data analysis

Wet stained gels were scanned using a GS-800™ Calibrated Imaging Densitometer (Bio-Rad, U.S.A) at a resolution of 600 dpi, bit depth 12. PDQuest software package v8.0.1 was used for image acquisition. Image analysis, spot detection, and quantification were done using the Progenesis SameSpots software package v4.1 (NonLinear Dynamics, Newcastle, UK), as previously detailed (Ferry et al. 2011; Guan et al. 2015). The threshold used to qualify differentially expressed spots was a Student t test, *p* value less than 0.05 and the false discovery rate (from q-values). Spot volumes (for each replicate spot in a group) for all groups were compared for each trial by ANOVA. Spots were determined to be significantly up- or downregulated when *p* value was < 0.05 (Tukey post hoc test). The molecular masses of protein spots on gels were determined by co-electrophoresis of a broad range molecular weight standard (Bio-Rad, U.S.A), and *pI* values for protein spots were estimated from the Immobiline DryStrip as per the manufacturer’s recommendations (GE Healthcare).

#### Liquid chromatography–mass spectrometry (LC–MS/MS) and database search

Selected protein spots were excised manually from the gel, and digested with sequencing grade-modified trypsin (Promega, USA) as described by Shevchenko et al. (1996). An ion-trap mass spectrometer (amaZon ETD, Bruker Daltonics, Bremen, Germany) equipped with an on-line nanospray source was used for mass spectrometry data acquisition. Solubilised peptides were fractionated on a nanoflow HPLC system (Thermo RSLCnano) before online analysis by electrospray ionisation (ESI) mass spectrometry on an amaZon speed ion ETD trap (Bruker Daltonics). Peptide separation was performed on a Pepmap C18 reversed phase column (LC Packings), using a 5–85% v/v acetonitrile gradient (in 0.5% v/v formic acid) run over 45 min at a flow rate of 0.2 µl/min. Mass spectrometric (MS) analysis was performed using a continuous duty cycle of survey. The mobile phase consisted of 0.1% v/v formic acid/H_2_O and the peptides were eluted at 200 nl/min. A survey scan was conducted in standard enhanced mode from m/z 400 to m/z 1600MS scan, followed by up to ten MS/MS analyses of the most abundant peptides, choosing the most intense multiply charged ions with dynamic exclusion for 120 s. The spectra that were obtained through the Bruker DataAnalysis 3.4 software from the MS/MS experiments were subsequently used for the confident identification of the proteins present in the mixture. Protein identifications were assigned using the Global Proteome Machine (GPM) search engine (www.thegpm.org) to interrogate protein sequences in the ENSEMBL Rattus Norvegicus database. The following settings were applied: method, ion trap (4 Da); cleavage site, trypsin [RK]|{P}; may missed cleavage, 1; complete modifications, carbamidomethyl; variable modification, oxidation (M); fragment mass error, 0.4 Da; parent mass error, ± 0.3 Da; fragment type, monoisotopic; maximum parent charge, 4. Only identification with log(e) values ≤ − 3 were regarded as significant hits regardless of the number of peptides.

### Statistical analysis

Significant differences were tested using the parametric analysis of variance (one-way ANOVA) and the nonparametric analysis of variance (Kruskal–wallis test), followed by Tukey Test. The IBM SPSS statistics 20 and Minitab 16 programs were used to perform statistical analyses. Differences were considered to be statistically significant when the *p* value was < 0.05 and highly significant when the *p* value was < 0.001.

## Results

No mortality occurred and there were no changes noted in terms of behaviour, activity, posture, gait, or external appearance for any of the different treatments for the duration of the two feeding studies. All animals in all treatment groups were healthy and appeared normal throughout the course of the study.

### Effects on weight gain, food consumption and feed conversion efficiency (FCE) of male Wistar rats

#### 7-Day rat feeding study

**Fig. 1:**
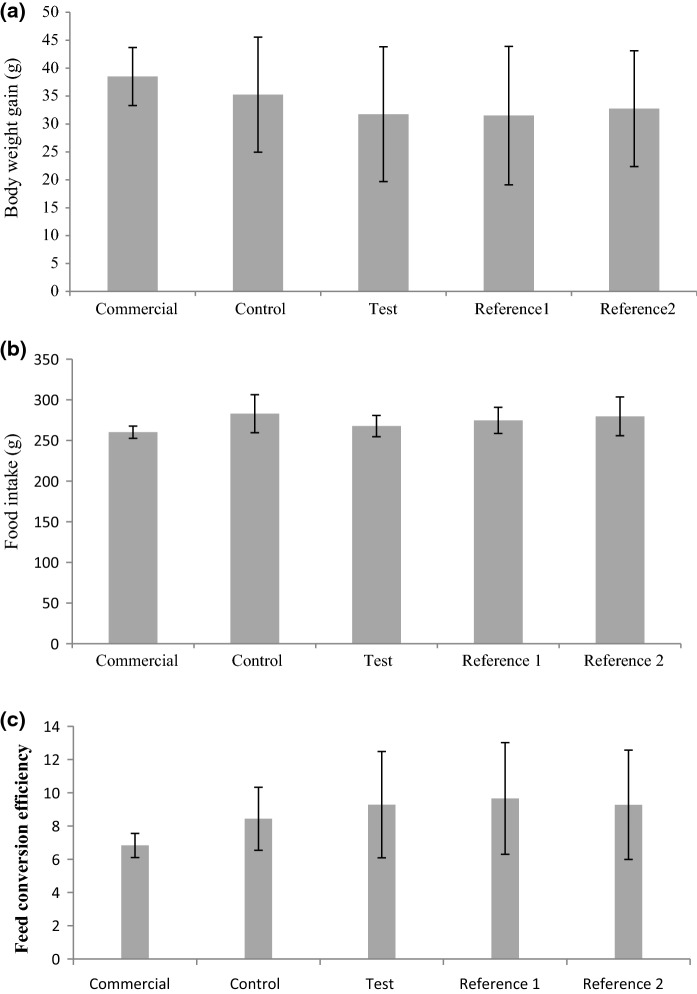
Performance of Wistar male rats fed different corn-based diets in 7-day feeding studies: **a** change in body weight gain (g); **b** food consumption (g); **c** feed conversion efficiency (FCE) over trial period. Values presented are ± SD; n = 4. Diets: Commercial, Purina rodent chow; Control, non-transgenic parental variety from which MON810 was derived; Test, transgenic *Bt* corn grain (MON810); Reference 1, Garst 8450; Reference 2, Golden Harvest H8920 (as detailed in Table S1 and S2)

Body weight gain was measured daily over the 7-day period. Although there were small differences between the different treatment groups, these were not significant (Fig. 1a; Table S3; *p* = 0.859) and were within the normal limits for rats of this strain, age and sex. Those fed the commercial Purina chow gained more weight and those fed Reference diet 1 gained the least weight, whilst rats fed the parental near isogenic line (Control group) gained more weight compared to those fed MON810 (Test group), but these differences were small and non-significant. Similarly, whilst there were small differences in food intake at the end of the 7-day period, this being greatest on the control diet (283 g) compared to the Test group (267.8 g), again this was not significant between the five groups (Fig. 1b; *p* = 0.695). These results are reflected in the calculated feed conversion efficiency (FCE) with those fed Purina chow having the lowest FCE (i.e. considered the most efficient users of feed); but this also was not significant between the five diets (Fig. 1c, Table S4; *p* = 0.600).

#### 28-Day rat feeding study

**Fig. 2:**
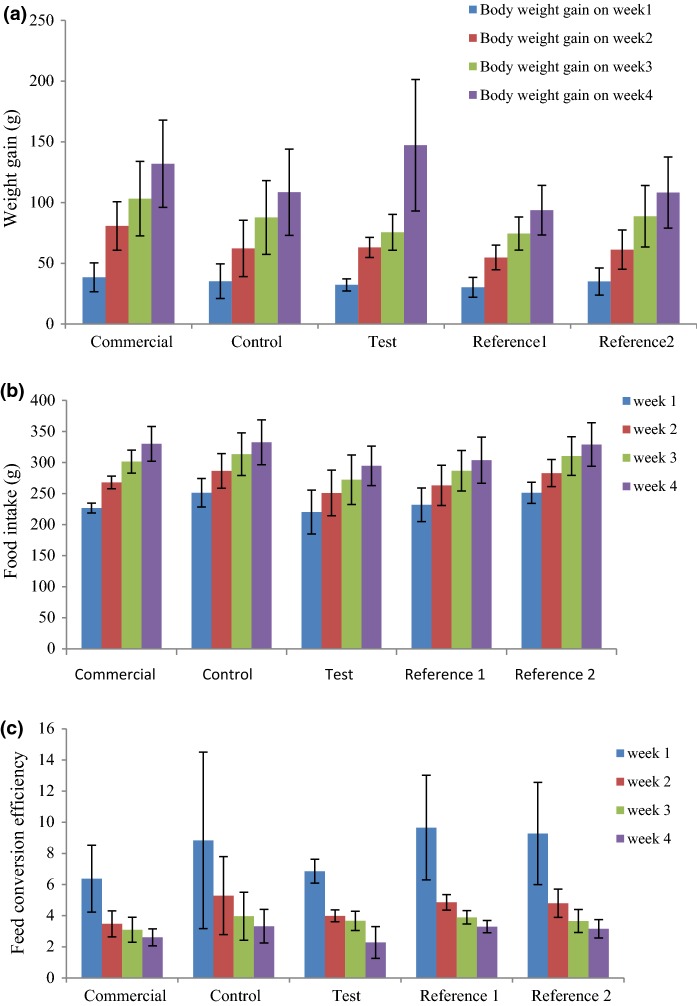
Performance of Wistar male rats on a weekly basis fed different corn-based diets in 28-day feeding studies: **a** change in body weight gain (g); **b** food consumption (g); **c** feed conversion efficiency (FCE) over trial period. Values presented are ± SD; n = 4. Diets: Commercial, Purina rodent chow; Control, non-transgenic parental variety from which MON810 was derived; Test, transgenic *Bt* corn grain (MON810); Reference 1, Garst 8450; Reference 2, Golden Harvest H8920 (as detailed in Table S1 & S2)

There was no significant differences in body weight gain of rats between the different treatment groups after 28 days (F4,15 = 1.35, *p *= 0.297 after 4 weeks). The residuals were not significantly different to a normal distribution (*p *= 0.682). The greatest increase was seen in the rats fed the Test diet (MON810), which was 35.7% greater than its near isogenic parental line (Fig. 2a; Table S5), followed by the commercial rodent diet. Food intake, calculated on a weekly basis, was the greatest on the control diet (332.5 g on week 4) compared to 294.75 g for the test group, however these differences between treatment groups were not significant (Fig. 2b; *p* = 0.450). The Feed conversion efficiency value was lowest for the Test group. However, as with all other parameters measured, differences in FCE between the five treatment groups were not significant (Fig. 2 c; Table S6; *p* = 0.270).

### In vivo effects on differential gene expression in the epithelial cells of the small intestine of male Wistar rats

**Fig. 3:**
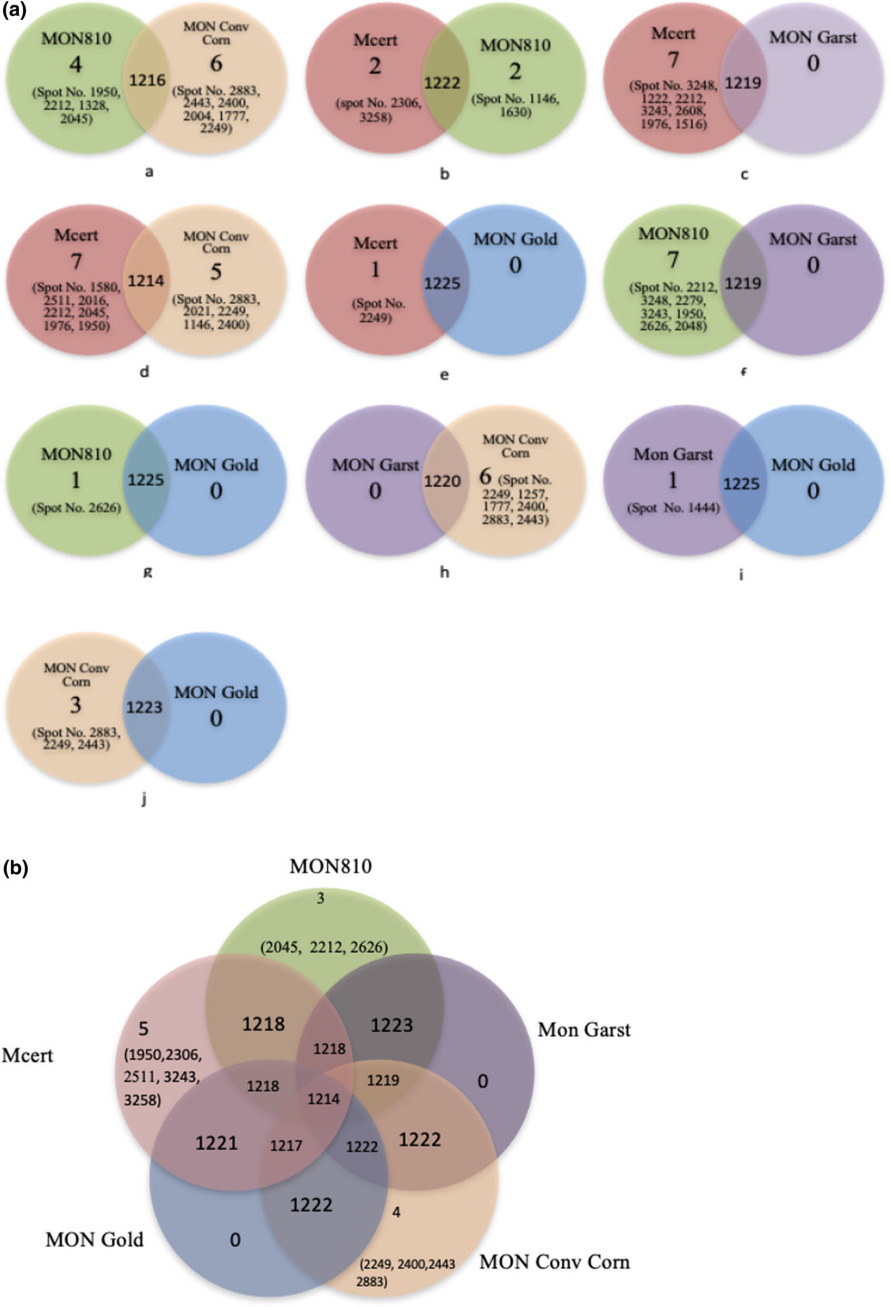
7-Day rat feeding studies: **a** two-set venn diagrams and **b** five-set venn diagrams comparing the changes in the differentially expressed protein spots and up-regulated spot number when rats were fed different corn-based diets. Diets: Commercial, Purina rodent chow; Control, non-transgenic parental variety from which MON810 was derived; Test, transgenic *Bt* corn grain (MON810); Reference 1, Garst 8450; Reference 2, Golden Harvest H8920 (as detailed in Table S1 & S2)

**Fig. 4:**
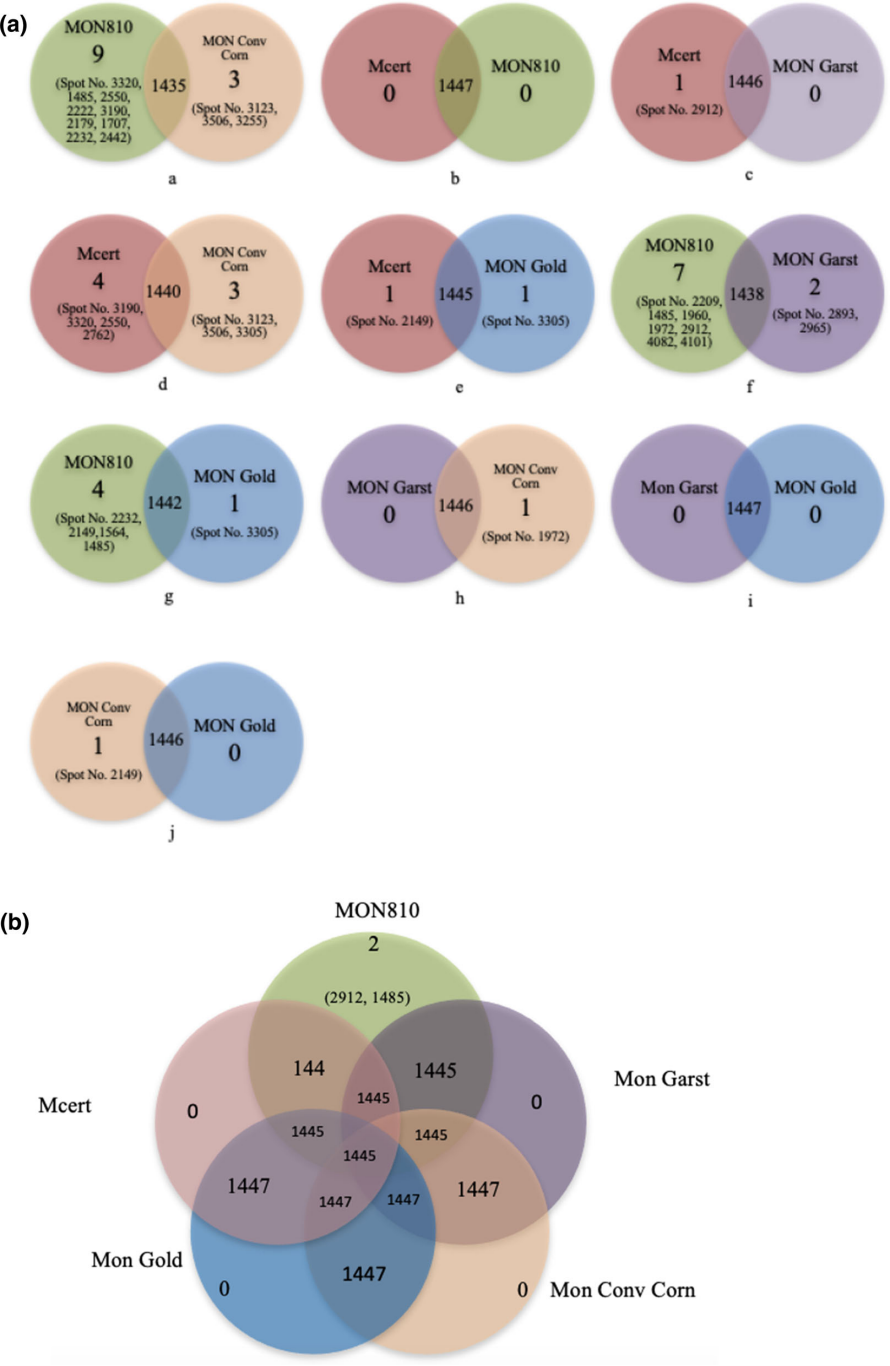
28-Day rat feeding studies: **a** two-set venn diagrams and **b** five-set venn diagrams comparing the changes in the differentially expressed protein spots and up-regulated spot number when rats were fed different corn-based diets. Diets: Commercial, Purina rodent chow; Control, non-transgenic parental variety from which MON810 was derived; Test, transgenic *Bt* corn grain (MON810); Reference 1, Garst 8450; Reference 2, Golden Harvest H8920 (as detailed in Table S1 & S2)

To obtain a better understanding of the underlying molecular responses in mammals to MON810, differential gene expression in the epithelial cells of the small intestine of rats fed formulated diets containing MON810, its near isogenic parental line, two conventional corn varieties, and a corn-based control diet were investigated using comparative proteomic profiling. Two-way (Figs. 3a, 4a) and five-way (Figs. 3b, 4b) comparisons of the differentially expressed protein spots were made between the different treatment groups both for the 7-day and 28-day studies. A Student t test *p* value less than 0.05 and the false discovery rate (FDR, from q-values) were used to qualify differentially expressed protein spots.

#### 7-Day rat feeding study

Comparison of the proteome maps for the IECs of rats fed MON810 with those fed the near isogenic parental line (Control; designated Mon Conv) identified 1216 protein spots in common between the two groups, which were expressed at similar levels. However, four protein spots (Spot No. 1950, 2212, 1328, 2045) were shown to be upregulated in response to the MON810 diet, whereas six (Spot No. 2883, 2443, 2400, 2004, 1777, 2249) were upregulated in the control fed rats. LC–MS/MS analysis identified two of these upregulated (2.4-fold) protein spots in the MON810 group to be stress-related proteins (catalase and 60 kDa heat shock protein); no stress related proteins were upregulated in the Control group (Fig. 3a; Table 1). Similar trends were also identified when the MON810 group was compared with the other groups. When compared with the commercial rodent diet 1222 protein spots were expressed at similar levels, with both groups exhibiting significant upregulation of only two protein spots each. Those upregulated in the MON810 group (spot No. 1146, 1630) encoded three stress-related proteins (catalase, 60 kDa heat shock protein and stress-induced phosphoprotein 1), but again no-stress related proteins were upregulated in the commercial rodent diet group (Spot No. 2306 and 3258). Comparison between the MON810 group and the Reference diet 1 (Mon Garst) group showed that of the seven protein spots (Spot No. 2212, 3248, 2279, 3243, 1950, 2626, 2048) upregulated in the Test group, three were classified as stress-related proteins, (thioredoxin-dependent peroxide reductase, peroxiredoxin-6 and LDLR chaperone MESD precursor). No proteins appeared to be upregulated in this Reference group. When compared with the Reference Group 2 (Mon Gold), again the only upregulated proteins were in the Test group, but in this instance there was only one (Spot No. 2626), which was identified as representing two stress-related proteins, (peroxiredoxin-6 and LDLR chaperone MESD precursor); however, the level of upregulation was only 1.2-fold.

**Table 1:** Seven-day rat feeding studies: functional classification of differentially expressed proteins in the epithelial cells of the small intestine of rats in response to feeding on: conventional diet (Mon Conv Corn group) formulated to contain approximately 33% control corn grain; test diet (MON 810 group) formulated to contain the test corn grain at approximately 33%; reference diets (Mon Garst and Mon Gold groups) formulated to contain the references corn grain at approximately 33% and commercial Purina rodent chow (Mcert group; purchased from Purina Mills Inc) which contains approximately 33% corn

Molecular function	Protein ID	Spot no.
Structural molecule activity	Prelamin-A/C	1146, 1630,
	Actin, cytoplasmic 1 Actin, cytoplasmic 1	2016, 2021
	Actin, gamma-enteric smooth muscle	2021
	Beta-actin-like protein 2	2021
Chaperones	60 kDa heat shock protein, mitochondrial	1630, 1580, 1328
	Calnexin precursor	1222
	Calreticulin precursor	1976
	T-complex protein 1 subunit zeta	1146
	T-complex protein 1 subunit beta (TCP-1-beta) (CCT-beta)	1444
	LDLR chaperone MESD Precursor (Mesoderm development candidate 2) (Mesoderm development protein)	2626
	Protein DJ-1 (Parkinson disease protein 7 homolog)	2608
Growth factor	Mesencephalic astrocyte-derived neurotrophic factor Precursor (Protein ARMET) (Arginine-rich protein)	2883
Oxidoreductase	Aldehyde dehydrogenase X, mitochondrial precursor	1516
	Hydroxyacyl-coenzyme A dehydrogenase, mitochondrial	2249
	D-beta-hydroxybutyrate dehydrogenase, mitochondrial Precursor (BDH) (EC 1.1.1.30) (3-hydroxybutyrate dehydrogenase)	2249
	Electron transfer flavoprotein-ubiquinone oxidoreductase, mitochondrial	1146
	Glyceraldehyde-3-phosphate dehydrogenase	2045, 2048
	Malate dehydrogenase, mitochondrial	2004
	Dihydrolipoyl dehydrogenase, mitochondrial Precursor (EC 1.8.1.4) (Dihydrolipoamide dehydrogenase)	1328, 1444
	UDP-glucose 6-dehydrogenase (UDP-Glc dehydrogenase) (UDP-GlcDH) (UDPGDH) (EC 1.1.1.22)	1328, 1444
	Methylmalonate-semialdehyde dehydrogenase, mitochondrial	1328
	Pyridine nucleotide-disulfide oxidoreductase domain-containing protein 2	1257
	Succinate dehydrogenase iron-sulfur subunit, mitochondrial Precursor (EC 1.3.5.1) (Iron-sulfur subunit of complex II)	2443
	Glutamate dehydrogenase 1, mitochondrial	1444
	Aldehyde dehydrogenase X, mitochondrial precursor	1516
	4-trimethylaminobutyraldehyde dehydrogenase (TMABADH) (EC 1.2.1.47)	1516
	Aldehyde dehydrogenase, mitochondrial Precursor (EC 1.2.1.3) (ALDH class 2) (ALDH1) (ALDH-E2)	1516, 1580
Antioxidant	Thioredoxin-dependent peroxide reductase, mitochondrial Precursor (EC 1.11.1.15) (Peroxiredoxin-3) (PRX-3) (PRx III)	3243
	Peroxiredoxin-6 (EC 1.11.1.15) (Antioxidant protein 2) (1-Cys peroxiredoxin) (1-Cys PRX)	2626
Electron transport	Cytochrome b-c1 complex subunit 2, mitochondrial Precursor (Ubiquinol-cytochrome-c reductase complex core protein 2) (Core protein II) (Complex III subunit 2)	1777
	Electron transfer flavoprotein subunit beta (Beta-ETF)	2608
	Electron transfer flavoprotein subunit alpha, mitochondrial Precursor (Alpha-ETF)	2279
Protein disulfide isomerase activity	Protein disulfide-isomerase A3	1444, 1516, 1580, 1630
Kinase	UMP-CMP kinase (EC 2.7.4.14) (Cytidylate kinase)	2608
Initiation factor in Protein biosynthesis	Eukaryotic translation initiation factor 3 subunit I (eIF3i) (Eukaryotic translation initiation factor 3 subunit 2) (eIF-3-beta)	2016
Chloride channel regulator activity	Na(+)/H(+) exchange regulatory cofactor NHE-RF1	2016
Transferase	N-acetyl-d-glucosamine kinase (N-acetylglucosamine kinase) (EC 2.7.1.59) (GlcNAc kinase)	2016
	3-Ketoacyl-CoA thiolase, mitochondrial	1777, 3248
	Phosphoglycerate kinase 1	1777
	Creatine kinase U-type, mitochondrial	1777
	Succinyl-CoA: 3-ketoacid-coenzyme A transferase 1, mitochondrial Precursor (EC 2.8.3.5)	1328
	Pyruvate kinase isozymes M1/M2	1257
	Dihydrolipoyllysine-residue succinyltransferase component of 2-oxoglutarate dehydrogenase complex, mitochondrial	2443
Porin	Voltage-dependent anion-selective channel protein 2 (VDAC-2) (Outer mitochondrial membrane protein porin 2) (B36-VDAC)	2212, 2279
	Voltage-dependent anion-selective channel protein 1	2249
Response to stress (co-chaperone)	Stress-induced-phosphoprotein 1	1146
Ribonucleoprotein	Heterogeneous nuclear ribonucleoprotein L isoform b	1146
	Heterogeneous nuclear ribonucleoproteins A2/B1 (hnRNP A2/B1)	2279, 2400
	Heterogeneous nuclear ribonucleoprotein H	1580
	Heterogeneous nuclear ribonucleoprotein H2 (hnRNP H2)	1580
	ErbB3-binding protein 1	1630
Peroxidase	Catalase	1146, 1257, 1328
Repressor	Coiled-coil-helix-coiled-coil-helix domain containing protein 3	2400
Muscle protein	Tropomyosin beta chain	1976
	Tropomyosin alpha-1 chain	1976, 2021, 3258
	Tropomyosin alpha-3 chain	2306
Serine-type endopeptidase inhibitor activity and Tumor suppressor	Serpin B5 (Peptidase inhibitor 5) (PI-5) (Maspin)	1950
Hydrolase	3′(2′),5′-bisphosphate nucleotidase 1 (EC 3.1.3.7)	1950
Translation elongation factor activity	Elongation factor 2	1950
Developmental protein	THO complex subunit 6 homolog	2048
Ligase	UPF0027 protein C22 or f28 homolog (p55)	1328
Mitosis	Centrosomal protein of 55 kDa	1328
Protease	Proteasome subunit alpha type-7 (EC 3.4.25.1) (Proteasome subunit RC6-1)	2443
	Cytosol aminopeptidase (EC 3.4.11.1) (Leucine aminopeptidase 3) (LAP-3) (Leucyl aminopeptidase) (Proline aminopeptidase) (EC 3.4.11.5) (Prolyl aminopeptidase)	1444
ATP binding	ATP synthase subunit alpha, mitochondrial	1444, 2443
Calcium ion binding/Transfer, Carrier protein	Annexin A11	1444
	Annexin A2	2045
DNA repair	PAX interacting (with transcription-activation domain) protein 1	2511
Epidermal growth factor receptor binding	Growth factor receptor-bound protein 2	2626

Not only were stress-related proteins identified in rats from the Test group when compared to the other treatments, but they were also shown to be upregulated when comparing the other diets with one another (Fig. 3a; Table 1). For example, when comparing the rodent diet (MCert) group with Reference 1, two stress-related proteins (thioredoxin-dependent peroxide reductase and protein DJ-1) were identified out of the seven upregulated proteins (Spot No. 3248, 1222, 2212, 3243, 2608, 1976, 1516) in the former group. When this group was compared with the Reference 2 group, there was only one upregulated protein and this again was in the rodent diet group; however, this protein was not associated with stress. The greatest number of differentially upregulated proteins was seen when comparing the rodent diet (MCert) group with the Control (near isogenic line of MON 810) group, where seven (Spot No. 1580, 2511, 2016, 2212, 2045, 1976, 1950) and five (Spot No. 2883, 2021, 2249, 1146, 2400) protein spots, respectively, were shown to be differentially upregulated. The 60 kDa heat shock protein was upregulated by threefold in the former group whilst stress-induced phosphoprotein 1 was upregulated in the latter. Comparisons between the Reference 1 group and Control group revealed that six protein spots were upregulated (Spot No. 2249, 1257, 1777, 2400, 2883, 2443) in the control and one of these, catalase, a stress-related protein was upregulated 2.6-fold. Comparison between the two reference groups only identified the stress-related protein T-complex protein 1 subunit beta as being upregulated.

In contrast to all the above two-way comparisons, no stress-related proteins were identified to be upregulated when the Control group was compared to the Reference 2 group. Whilst the expression of 1223 protein spots was not significantly different to one another, three (Spot No. 2883, 2249, and 2443) were upregulated in the control diet, but none of these were associated with stress-related proteins.

Analysis of the data in a five-way comparison showed that far fewer proteins were differentially expressed, this being five, four, and three protein spots for the rodent diet (of which one was the stress-related protein thioredoxin-dependent peroxide reductase), control and MON810 (of which two were the stress-related proteins LDLR chaperone MESD precursor and peroxiredoxin-6), respectively. There was no differential expression in either of the two reference groups (Fig. 3b; Table 1). Full details of protein spots in common, and those upregulated, between the treatments, with their fold-change, are presented in Fig. 3a, b, Table S7. What is interesting to note was the similarity between the different treatments with the number of protein spots in common ranging from 1214 to 1225; this very small variance illustrates the robustness of the data.

#### 28-Day rat feeding study

Two-way and five-way comparisons were also carried out for the 28-day feeding studies. What is interesting to note was the overall similarity in the response at 7 days and 28 days in terms of differential regulation of stress-related proteins between different treatments. Overall, in two-way analyses, fewer proteins were differentially expressed and fewer of these were classified as stress-related (Fig. 4a, b; Table 2). Comparison of the MON810 group with its near isogenic line (Control) revealed that the number of upregulated protein spots in the former group had increased to nine (Spot No. 3320, 1485, 2550, 2222, 3190, 2179, 1707, 2232, 2442) whilst in the latter this number had decreased to three (Spot No. 3123, 3506, 3255); however the number of stress-related proteins in the Test group remained at two (stress-induced phosphoprotein 1 and peroxiredoxin-1), although these were different to those upregulated in the 7-day study; in this instance one stress-related protein, superoxide dismutase, was upregulated in the control group (Fig. 4a). In contrast to the 7-day study, no proteins were differentially expressed between the MON810 and rodent diet group in the 28-day study, with 1446 proteins exhibiting expression levels in common. When compared to Reference 1, and Reference 2, there were seven (Spot No. 2209, 1485, 1960, 1972, 2912, 4082, 4101) and four (Spot No. 2232, 2149, 1564, 1485) upregulated proteins, respectively and two (Spot No. 2893, 2965) and one (Spot No. 3305), respectively for the reference groups. Of those upregulated in the MON810 group vs Reference 1, two in the Test group were stress-related proteins (stress-induced phosphoprotein 1 and 60 kDa heat shock protein) and one in the comparator (T-complex protein 1 subunit beta). When compared to the Reference 2 group, only two stress-related proteins (both phosphoprotein 1) were upregulated in total, and both in the MON810 group. The only other stress-related protein to be upregulated between the other treatments in the two-way comparisons was peroxiredoxin-1 (2.8-fold) in the rodent diet when compared with the control diet (Fig. 4a; Table 2).

**Table 2:** Twenty eight-day rat feeding studies: functional classification of differentially expressed proteins in the epithelial cells of the small intestine of rats in response to feeding on: conventional diet (Mon Conv Corn group) formulated to contain approximately 33% control corn grain; test diet (MON 810 group) formulated to contain the test corn grain at approximately 33%; reference diets (Mon Garst and Mon Gold groups) formulated to contain the references corn grain at approximately 33% and commercial Purina rodent chow (Mcert group; purchased from Purina Mills Inc) which contains approximately 33% corn

Molecular function	Protein ID	Spot no.
Structural molecule activity	Lamin-A	1485, 1564
Chaperones	60 kDa heat shock protein, mitochondrial	1960
	T-complex protein 1 subunit beta (TCP-1-beta) (CCT-beta)	2893
Oxidoreductase	Isocitrate dehydrogenase subunit alpha, mitochondrial Precursor (EC 1.1.1.41)	3190
	Isocitrate dehydrogenase, mitochondrial	2222
	Isocitrate dehydrogenase cytoplasmic	2209, 4101
	Glyceraldehyde-3-phosphate dehydrogenase	4082
	Malate dehydrogenase, mitochondrial	4082
	Dihydrolipoyl dehydrogenase, mitochondrial Precursor (EC 1.8.1.4) (dihydrolipoamide dehydrogenase)	1707
	Superoxide dismutase, mitochondrial Precursor (EC 1.15.1.1)	3255
	Alcohol dehydrogenase (EC 1.1.1.2) (Aldehyde reductase) (Aldo–keto reductase family 1 member A1) (3-DG-reducing enzyme)	2442
Antioxidant/peroxidase	Peroxiredoxin-1 (EC 1.11.1.15) (thioredoxin peroxidase 2) (thioredoxin-dependent peroxide reductase 2)	3190
Electron transport	Cytochrome b-c1 complex subunit 2, mitochondrial Precursor (Ubiquinol-cytochrome-c reductase complex core protein 2) (core protein II)	2232
	Electron transfer flavoprotein subunit beta (Beta-ETF)	2912
Protein disulfide isomerase activity	Protein disulfide-isomerase A3	2149, 1707, 1960, 1972
Kinase	Adenylate kinase 2, mitochondrial (AK 2) (EC 2.7.4.3) (ATP-AMP transphosphorylase 2)	2912
	Phosphoglycerate kinase 1	2232
Transferase	Ornithine aminotransferase, mitochondrial Precursor (EC 2.6.1.13) (Ornithine–oxo-acid aminotransferase)	2550, 2179
	3-Ketoacyl-CoA thiolase, mitochondrial	2222
Response to stress (co-chaperone)	Stress-induced-phosphoprotein 1	1485, 1564
Ribonucleoprotein	Heterogeneous nuclear ribonucleoprotein A3 (hnRNP A3)	4082
	heterogeneous nuclear ribonucleoprotein U	2550
	40S ribosomal protein S3	2762
Repressor	Chromobox homolog 3	3305
Muscle protein	Actin, gamma-enteric smooth muscle	3506
	Actin, cytoplasmic 1 Actin, cytoplasmic 1, N-terminally processed	3506
Hydrolase	6-Phosphogluconolactonase (6PGL) (EC 3.1.1.31)	2965
	Acyl-coenzyme A thioesterase 2, mitochondrial	4101
Protease	Proteasome subunit alpha type-4 (EC 3.4.25.1) (proteasome component C9)	2912
	Cytosol aminopeptidase (EC 3.4.11.1) (Leucine aminopeptidase 3) (LAP-3) (Leucyl aminopeptidase) (proline aminopeptidase) (EC 3.4.11.5) (prolyl aminopeptidase)	2442
	Lactotransferrin; lactoferrin; EC 3.4.21.	2893
NADH dehydrogenase (ubiquinone) activity	NADH dehydrogenase (ubiquinone) 1 beta subcomplex, 10	3190, 3123
	NADH dehydrogenase (ubiquinone) 1 beta subcomplex, 9	3320
Regulation of microtubule-based process	Protein MEMO1	2762
Protein binding	Calcyclin-binding protein	2912
Ligase	Tyrosyl-tRNA synthetase, cytoplasmic (EC 6.1.1.1) (Tyrosyl–tRNA ligase) (TyrRS)	1707
Actin capping	Gelsolin precursor (actin-depolymerizing factor) (ADF) (Brevin)	2209
Lyase	Alpha-enolase (EC 4.2.1.11) (2-phospho-d-glycerate hydro-lyase) (non-neural enolase) (NNE) (enolase 1)	1960
DNA binding	DNA-directed RNA polymerases I, II, and III subunit RPABC1 (RNA polymerases I, II, and III subunit ABC1) (DNA-directed RNA polymerase II subunit E)	2965

Comparisons of differentially expressed proteins between all five treatment-groups identified that only two protein spots were upregulated, both in the MON810 group (Spot No. 2912, 1485), one of which was stress-induced phosphoprotein 1 (upregulated 3.1-fold). Full details of protein spots in common, and those upregulated, between the treatments, with their fold-change, are presented in Fig. 4a, b, Table S8. There were slightly more proteins visualized on the proteome maps for the 28-day study, with the variance between treatments being even smaller (number of protein spots ranging from 1445 to 1447).

To assess the functional relevance of changes in the differentially expressed proteins identified, proteins were aligned according to their molecular functions, based on information provided by the online resource UniProt classification system. Some proteins were annotated manually, based on literature searches and closely related homologues. There were 28 groups for the 7-day study and 21 groups for the 28-day study (Figs. S1, S2).

## Discussion

Corn lines used for the present study were grown simultaneously in neighbouring fields, under the same cultural and climatic conditions, thus eliminating, or at least reducing, environmental variables as far as reasonably practical. Three reference lines were used to determine whether any potential changes that may occur with the consumption of MON810 corn lay within the range for several different unmodified reference corn varieties. Compositional analysis of the transgenic and non-transgenic corn varieties confirmed that the diets fed to the rats were similarly balanced in terms of nutrition and energy content.

Monitoring appearance, behaviour, body weight gain and food consumption is a sensitive indicator of overall animal health. In the present study no adverse clinical signs or behavioural effects were observed. There were no significant differences in absolute body weights, body weight gains, food consumption or feed conversion efficiency between animals fed MON810 compared to animals fed diets containing grain from its near isogenic line (Control), reference varieties or the commercial rodent diet control. Whilst a small number of animals may affect the statistical power regarding the detection of subtle differences, in line with requirements governing the responsible use of animals for experimentation (Balls and Fentem 2008), four rats per group is considered to be the minimum, but acceptable, number used by the industry in such studies. The comparable responses of rats fed MON810 to those fed control grain are in agreement with those of Hammond et al. (2006) for MON810 and its near isogenic parental line, leading these authors to hypothesise that substantial safety margins exist for human consumption of MON810. It is noteworthy that similar studies with pigs, as a human model, failed to demonstrate any toxic effects on either growth or the intestinal flora as a consequence of feeding on MON810 in either short-term or long-term studies (Buzoianu et al. 2012; Walsh et al. 2012). This lack of observed toxicity has also been reported for *Bt* rice in both 28-day and 90-day studies (Kroghsbo et al. 2008; Yuan et al. 2013).

Whilst numerous studies have demonstrated the lack of toxicity of MON810 to mammals, to the best of our knowledge no previous studies have investigated any potential underlying molecular responses at the proteome level. As the intestine is one of the common targets of psychological and physical stress (Soderholm and Perdue 2001), any impact on the proteome of the epithelial cells of the small intestine of rats in response to different diets was investigated. The changes for all treatments are presented, but the focus here is on the differential regulation of stress-related proteins in response to ingestion of MON810 with its direct comparator, the non-transgenic near-isogenic parental line (Control). These stress-related proteins are good indicators of a range of different types of physical and psychological stress, including first exposure to new food components prior to immune recognition/tolerance, infection, inflammation, exercise, exposure of the cell to toxins, starvation, hypoxia, water deprivation or anxiety.

Pairwise comparisons showed that, with the exception of rats fed MON Gold (Reference 2) where no detectable proteins were differentially expressed in the S.I. epithelial cells, the majority of proteins that were expressed in response to consumption of the different diets were related to metabolism, such as lipid and carbohydrate metabolism, and protein biosynthesis; the number ranging from seven to only one. Comparison between rats fed MON810 and its near isogenic control (Mon Conv Corn group) showed that two stress-related proteins (catalase and 60 kDa heat shock protein) were upregulated in rats fed MON810, with no stress-related proteins being upregulated in the latter group. This 60 kDa heat shock protein is known to be expressed in response to a wide variety of physiological and environmental stresses, acting as a molecular chaperone for nascent and stress-accumulated misfolded proteins, or mediating immunological functions, thus exerting a protective role (Nakamura et al. 1991; Otaka et al. 1997; Schoeniger et al. 1994). Cells from virtually all organisms respond to a variety of stresses by the rapid synthesis of a highly conserved set of polypeptides termed heat shock proteins (HSPs). The precise functions of HSPs are unknown, but there is considerable evidence that they are essential for survival at both normal and elevated temperatures. HSPs play an important role in both normal cellular homeostasis and the stress response suggesting that they may be important modifying factors in cellular responses to a variety of physiologically relevant conditions such as hyperthermia, exercise, oxidative stress, metabolic challenge, and aging (Kregel 2002). Expression of HSPs is thought to contribute to the higher capability of young rats to cope with xenobiotics and stress conditions (Malatesta et al. 2008). Takada et al. (2010) examined the effects of overexpression of HSP60 and HSP70 on hydrogen peroxide induced cell damage using rat S.I. cells (IEC-6). They found that the viability was significantly higher in both HSP60- and HSP70-overexpressing cells compared with that of control cells and concluded that HSP60 plays an important role in protecting these cells from H_2_O_2_-induced cell injury. The other stress-related protein, which was upregulated in rats fed MON810, was catalase. Tissues are normally protected from oxidative damage by the presence of enzymes such as catalase, which is an oxidative stress response protein occurring in almost all aerobically respiring organisms and serves to remove the hydrogen peroxide formed in the cells to protect them from the toxic effects of this compound (Takeuchi et al. 1995). However, this upregulation of stress-response proteins was not unique to rats fed MON810 since pair-wise comparisons showed that rats fed other corn-based diets in the present study also resulted in differential expression of stress proteins. For example, comparison between rats fed MCert corn with Mon Garst corn revealed that two stress-related proteins (Thioredoxin-dependent peroxide reductase and protein DJ-1) were upregulated in the MCert corn-fed rats, but none were upregulated in Mon Garst corn-fed rats, whilst comparison between MCert corn-fed rats and Mon Conv corn-fed rats showed that 1 stress-related protein (60 kDa heat shock protein) was upregulated in the former and 1 stress related protein (stress-induced phosphoprotein 1) was upregulated in the latter. Importantly, the 7-day rat feeding study confirmed the upregulation of a few stress-related proteins not only in MON810 corn-fed rats but also in the near isogenic control corn-fed rats, reference corn variety-fed rats (Mon Garst group) and commercial corn-fed rats. Thus, the subsequent up-regulation of these stress-related proteins in the rat small intestine is not unique to the GM corn diet, but was also shown to occur in the non-transgenic corn lines.

Consistent with trends observed in the 7-day trial, the majority of differentially expressed proteins between MON810-fed rats and those fed its near isogenic line (Control) in the 28-day study were related to metabolism, although, again, some stress-response proteins were also shown to be differentially expressed. Interestingly, the same results were found with rats fed the other commercial corn varieties. Comparison of differentially expressed proteins between rats fed MON810 with those fed the control diet showed that two stress-related proteins (stress-induced phosphoprotein 1 and peroxiredoxin-1) were upregulated in rats fed MON810 and one stress related protein (superoxide dismutase) was upregulated in the control. Peroxiredoxin-1 and superoxide dismutase are both antioxidant defence enzymes. Peroxiredoxin-1 is involved in the redox regulation of cells, such as reducing peroxides (Zhang et al. 2003). Peroxiredoxin proteins have conserved cysteine residues that participate in oxidoreductive reactions and protect macromolecules from oxidative damage and are known to protect cells from apoptosis by reducing oxidative damage (Ishii et al. 2000). Superoxide dismutase destroys radicals which are normally produced within the cells and which are toxic to biological systems (MacMillan-Crow and Thompson 1999). Interestingly, comparison between rats fed the different non-transgenic corn lines similarly showed differential expression of stress-related proteins. For example, comparison between differentially expressed proteins between rats fed MCert corn and Mon Conv Corn showed that 1 stress-related protein (peroxiredoxin-1) was upregulated in rats fed the MCert corn.

As reported for the 7-day study, the 28-day rat feeding trial also demonstrated the upregulation of stress-related proteins both in rats fed the transgenic (MON810) corn and those fed the non-transgenic corn varieties. These results highlight the importance of including other conventional varieties when evaluating the effects of a GM ingredient and suggest that these stress-related proteins found in MON810-fed rats are not directly related to the consumption of MON810 corn diet, but are due to normal physiological variation. We can conclude that the in vivo effects of these different corn varieties on the proteome of the epithelial cells of the S.I. are negligible, with only one stress-related protein being upregulated in MON810-fed rats when the five different groups were compared to each other. The difference in the number of these stress-related proteins is likely to be due to the physiological differences between rats. The only other study to have used proteomics to investigate the stress response in rodents was that of Zhang et al. (2003) who studied the differentially expressed proteins of gamma-ray irradiated mouse intestinal epithelial cells. The majority of differentially expressed proteins are involved in metabolism, anti-oxidation, protein post-translational processes, and signal transduction, although upregulation of three oxidative stress response proteins (peroxiredoxin I, glutathione S-transferase P2, and antioxidant protein 2) were also reported.

The mucosal response observed in this study is likely to be a natural physiological response to the exposure to new diet components, particularly given the fact that all rats were initially reared, and acclimatised, on a basal diet that was slightly different to that of the treatments. Thus the gut mucosal response in terms of differential gene expression, albeit mild and non-significant, is likely due to the recognition of new components in the diet and the inevitable immune sequelae. Upregulation of stress-response proteins is not necessarily an undesirable effect and indeed is often advantageous. Physiological and psychological stress is a common event in normal daily life and a mild stress can be beneficial because it improves physiological functions in the body, such as a mild increase in noradrenalin in the blood, thus improving circulation, and increased secretion of thyrotrophic releasing hormone, which facilitates metabolism (Gardner 2001). The intestine is one of the common targets of stress and acts as a barrier between the external and internal environment (Nagler-Anderson 2001). As discussed above, HSPs are involved in the cellular response to a variety of environmental stresses like heat, cold and oxygen deprivation (Trautinger 2001), playing a role in protein-folding and trafficking, e.g. the trafficking of peptides to the cell surface to help the immune system recognize diseased cells (Fehrenbach and Northoff 2001; Latchman 2001). Thus the expression of these stress-related proteins in the epithelial cells of the rat S.I. observed in the present study does not indicate any adverse effects, and is not unique to rats fed MON810 corn but represents normal biological variability. These findings are consistent with recent complementary studies by Coumoul et al. (2018) who were unable to detect biomarkers of adverse health effect in rats following consumption of the same *Bt*-corn event (MON810) or, indeed, genetically modified herbicide tolerant corn (NK603), compared to consumption of their near-isogenic controls using both transcriptomics and metabolomics. Our findings are also consistent with the findings of Wang et al. (2013) who demonstrated that transgenic *Bt* rice, expressing the same Cry protein as expressed in MON810, was not toxic to female Swiss rats and had no effect on a range of stress-related enzymes including catalase, and superoxide dismutase in heart, liver, spleen, brain, kidney and ovary. Furthermore, they also showed that transcript levels for these two stress-related enzymes were not affected by the presence of Cry1Ab in the diet.

In conclusion, we identified no biomarkers of exposure or effects in the epithelial cells of the small intestine in rats, which represent comparable tissues to those targeted by *Bt* in insects. Furthermore, we conclude that the observed differences in differentially expressed proteins, including stress-related proteins, following consumption of transgenic corn (MON810), commercial varieties and controls are consistent with normal physiological variation. The observed proteomic variations are similarly unremarkable for rats of this strain and age and were not associated with any adverse health effects. These data can potentially be extrapolated to man and livestock, given the general acceptance of rats as a surrogate animal model for other mammalian species.

## Electronic supplementary material

Below is the link to the electronic supplementary material.

Supplementary material 1 (DOCX 44 kb)
