# Supplementary material for: A proteomic-based approach to study underlying molecular responses of the small intestine of Wistar rats to genetically modified corn (MON810)
Source: Transgenic Res. 2019 Jun 6;28(5):479–98. doi: 10.1007/s11248-019-00157-y (PMC6848250; doi:10.1007/s11248-019-00157-y)
Supplement: Supplementary file 1 — Supplementary material 1 (DOCX 44 kb) [file 11248_2019_157_MOESM1_ESM.docx]

**Supplementary Figure Legends**

**Figure S1:** Seven Day-Rat Feeding Studies: Functional classification of differentially expressed proteins in the epithelial cells of the small intestine of rats in response to feeding on: conventional diet (Mon Conv Corn group) formulated to contain approximately 33% control corn grain; test diet (MON 810 group) formulated to contain the test corn grain at approximately 33%; reference diets (Mon Garst and Mon Gold groups) formulated to contain the references corn grain at approximately 33% and commercial Purina rodent chow (Mcert group; purchased from Purina Mills Inc) and contains approximately 33% corn. A total of 88 proteins were identified representing 28 major functional categories. These were classified as: oxidoreductases (19; 22%), chaperones (9; 10%), are transferases (8; 9%), 6 (7%) protein involved in structural molecule activity and 6 (7%) are ribonucleoproteins.

**Figure S2:** Twenty Eight-Day Rat Feeding Studies: Functional classification of differentially expressed proteins in the epithelial cells of the small intestine of rats in response to feeding on: conventional diet (Mon Conv Corn group) formulated to contain approximately 33% control corn grain; test diet (MON 810 group) formulated to contain the test corn grain at approximately 33%; reference diets (Mon Garst and Mon Gold groups) formulated to contain the references corn grain at approximately 33% and commercial Purina rodent chow (Mcert group; purchased from Purina Mills Inc) and contains approximately 33% corn. A total of 47 proteins were identified and these proteins fall within 21 major functional categories. 9 (19%) contain oxidoreductase activity, 2 (4%) are chaperones, 3 (6%) contained transferase activity, 2 (4%) contained structural molecule activity and 3 (6%) are ribonucleoproteins.

**Supplementary Figures**

**Fig S1**

**Fig S2**

**Supplementary Tables**

Table S1 Ingredient Composition (% dry wt) of experimental diets for 7-Day and 28-Day rat studies

| Ingredients | Test  (MON810) | Reference 1 (MON Garst 8450) | Reference 2 (MON Gold H8920) | Control  (MON Conv Corn) |
| --- | --- | --- | --- | --- |
| **Cereal products** (ground Corn, Ground Oats, Wheat Germ, Ground Wheat, Wheat Middlings) | 61 | 59.4 | 58.3 | 58.1 |
| **Vegetable Proteins** (Dehydrated Alfalfa Meal, Dried Beet Pulp, Ground Soybean Hulls, Dehulled Soybean Meal, Dried Brewers Yeast) | 26.6 | 27.8 | 30.3 | 29.7 |
| **Animal Proteins** (fish Meal, Acid Casein, Dried Whey) | 6.1 | 6.4 | 5.0 | 6.0 |
| **Energy Sources** (Animal Origin) | 0.0 | 0.0 | 0.0 | 0.0 |
| **Energy Sources**-All other (Soybean Oil, Cane Molasses) | 4.2 | 4.3 | 4.2 | 4.1 |
| Supplementation (vitamins, Major Minerals, Trace Minerals, Amino Acids) | 2.1 | 2.1 | 2.2 | 2.1 |

*All diets and the commercial chow (not shown) are closely based on the Certified Rodent LabDiet 5002. Control and reference grains (MON Garst and MON Gold) are from conventional varieties; only the test (MON810) diet is a Biotech crop.

**Table S2** Organisation of study groups

| Group | Treatment | Group diet Identification | 7-day Feeding Study; Males | 28-day Feeding Study; Males |
| --- | --- | --- | --- | --- |
| I | Conventional | Control | 4 | 4 |
| II | MON 810 | Test | 4 | 4 |
| III | Garst 8450 | Reference 1 | 4 | 4 |
| IV | Golden Harvest H8920 | Reference 2 | 4 | 4 |
| V | PMI Commercial diet | Commercial | 4 | 4 |

Rats in Group I were fed diet containing 33% corn from the non-transgenic parental variety from which MON810 was derived; Group II were fed the test diet containing 33% transgenic *Bt* corn grain (MON810); Groups III and IV were fed diet containing 33% ~33% conventional corn as a reference standard. Group V were fed commercial Purina rodent chow containing ~ 33% corn.

Table S3 7-Day Rat Feeding Studies: Differences in body weight gain on D8 compared to the control. Values are means ± SD.

| Group (n=4) | Body weight Gain on D8 | Difference with control (%) |
| --- | --- | --- |
| Commercial | 38.5 ±5.19 | 9.2 |
| Control | 35.25±10.30 | --- |
| Test | 31.75±12.06 | -9.9 |
| Reference1 | 31.5±12.39 | -10.6 |
| Reference2 | 32.75±10.37 | -7.1 |

Table S4 7-Day Rat Feeding Studies: Feed conversion efficiency (FCE)

| Group (n=4) | Group mean food intake in week 1 | Group mean weight gain in week 1 | Feed conversion efficiency | | |
| --- | --- | --- | --- | --- | --- |
| Commercial | 260.25 | 38.5 |  | 6.831±0.72 |  |
| Control | 283 | 35.25 |  | 8.4375±1.89 |  |
| Test | 267.75 | 31.75 |  | 9.28625±3.20 |  |
| Reference 1 | 274.75 | 31.5 |  | 9.656±3.35 |  |
| Reference 2 | 279.75 | 32.75 |  | 9.278±3.28 |  |

**Table S5** 28-Day Rat Feeding Studies: Differences in body weight gain on D8 compared to the control. Values are means ± SD.

| Groups (n=4) | Body wt: Week 1 | Body wt: Week 2 | Body wt: Week 3 | Body wt: Week 4 | Relative difference to control (%) |
| --- | --- | --- | --- | --- | --- |
| Commercial | **38.5**±11.90 | **80.75**±20.02 | **103.25**±30.73 | **132**±35.91 | 21.6 |
| Control | **35.25**±14.22 | **62.25**±23.22 | **87.75**±30.31 | **108.5**±35.51 | --- |
| Test | **32.25**±4.99 | **63**±8.28 | **75.5**±14.73 | **147.25**±54.10 | 35.7 |
| Reference1 | **30.25**±8.18 | **54.75**±10.14 | **74.5**±13.67 | **93.75**±20.43 | -13.6 |
| Reference2 | **35**±11.16 | **61.25**±16.15 | **88.75**±25.31 | **108.25**±29.34 | -0.23 |

**Table S6** 28-Day Rat Feeding Studies: Feed conversion efficiency (FCE)

| Group (n=4) | Week 1 | Week 2 | Week 3 | Week 4 |
| --- | --- | --- | --- | --- |
| Commercial | **6.3**8**±**2.14 | **3.47±**0.83 | **3.09±**0.80 | **2.61±**0.54 |
| Control | **8.83±**5.66 | **5.29±**2.50 | **3.96±**1.53 | **3.32±**1.07 |
| Test | **6.85±**0.76 | **3.99±**0.38 | **3.67±**0.61 | **2.27±**1.01 |
| Reference 1 | **9.65±**3.35 | **4.86±**0.49 | **3.89±**0.42 | **3.29±**0.39 |

Table S7: Identification of differentially expressed stress-related proteins from the epithelial cells of the small intestine of rats fed different maize based diets for 7-day.

The data represent two way comparisons as follows (the transgenic *Bt* maize MON810 vs its corresponding parental non-transgenic control (near isogenic) maize) and five way comparisons between all different maize based diets.

| **Spot No.** | | **Fold** | | **Experimental pI** | | **Experimental MW** | **Protein Description** | **Function and biological process** | | **rI** | | **log(e)** | | **Theoritical pI** | **Theoritical MW (KiloDaltons)** | | **Average Normalised Volumes** | | | | | |
| --- | --- | --- | --- | --- | --- | --- | --- | --- | --- | --- | --- | --- | --- | --- | --- | --- | --- | --- | --- | --- | --- | --- |
|  |  |  |  |  |  |  |  |  |  |  |  |  |  |  |  |  | **MON810** | | **Control** | | | |
| **1328** | | 2.4 | | 7.55 | | 57,752 | Catalase | Occurs in almost all aerobically respiring organisms and serves to protect cells from the toxic effects of hydrogen peroxide. Promotes growth of cells. | | 4 | | -24.6 | | 7.1 | 59.7 | | 2.17E+06 | | 8.89E+05 | | | |
|  |  |  |  |  |  |  | 60 kDa heat shock protein, mitochondrial | Implicated in mitochondrial protein import and macromolecular assembly. May facilitate the correct folding of imported proteins. May also prevent misfolding and promote the refolding and proper assembly of unfolded polypeptides generated under stress conditions in the mitochondrial matrix. | | 4 | | -22.4 | | 5.9 | 60.9 | |  |  |  |  |  |  |
| **Spot No.** | **Anova (p)** | | **Fold** | | **Experimental Ip/MW (MW in KiloDaltons)** | | **Protein Description** | **Function** | **rI** | | **log(e)** | | **Theoritical Ip/MW**  **(MW in KiloDaltons)** | | | **Average Normalised Volumes** | | | | | | |
|  |  |  |  |  |  |  |  |  |  |  |  |  |  |  |  | **Rodent diet** | | **MON810** | | **Reference 1 (Mon Garst)** | **Control** | **Reference 2 (Mon Gold)** |
| **2626** | 4.58E-04 | | 2.4 | | 6.18/26.990 | | LDLR chaperone MESD Precursor | Probably plays a role in facilitating the assembly of multimeric protein complexes inside the ER. | 10 | | -47 | | 5.5/25.2 | | | 3.62E+06 | | 3.75E+06 | | 2.05E+06 | 1.55E+06 | 3.14E+06 |
|  |  |  |  |  |  |  | Peroxiredoxin-6 | Involved in redox regulation of the cell. Can reduce H2O2 and short chain organic, fatty acid, and phospholipid hydroperoxides. May play a role in the regulation of phospholipid turnover as well as in protection against oxidative injury | 7 | | -33 | | 5.6/24.8 | | |  |  |  |  |  |  |  |
| **3243** | 0.002 | | 2.6 | | 6.33/28.246 | | Thioredoxin-dependent peroxide reductase | Involved in redox regulation of the cell. Protects radical-sensitive enzymes from oxidative damage by a radical-generating system. Acts synergistically with MAP3K13 to regulate the activation of NF-kappa-B in the cytosol. (Human) | 9 | | -16 | | 7.1/28.3 | | | 2.86E+06 | | 2.83E+06 | | 1.12E+06 | 1.13E+06 | 1.64E+06 |

log(e): the base-10 log of the expectation that any particular protein assignment was made at random (E-value).

rl: the number of unique peptide sequences associated with this protein assignment.

pI: isoelectric point for the intact gene product.

Table S8: Identification of differentially expressed stress-related proteins from the epithelial cells of the small intestine of rats fed different maize based diets for 28-day.

The data represent two way comparisons as follows (the transgenic *Bt* maize MON810 vs its corresponding parental non-transgenic control (near isogenic) maize) and five way comparisons between all different maize based diets

| **Spot No.** | | **Fold** | | **Experimental pI** | | **Experimental MW** | **Protein Description** | **Function and biological process** | | **rI** | | **log(e)** | | **Theoritical pI** | **Theoritical MW (KiloDaltons)** | | **Average Normalised Volumes** | | | | | |
| --- | --- | --- | --- | --- | --- | --- | --- | --- | --- | --- | --- | --- | --- | --- | --- | --- | --- | --- | --- | --- | --- | --- |
|  |  |  |  |  |  |  |  |  |  |  |  |  |  |  |  |  | **MON810** | | **Control** | | | |
| **1485** | | 3.1 | | 6.92 | | 73,807 | Stress-induced-phosphoprotein 1 | Mediates the association of the molecular chaperones HSC70 and HSP90 (HSPCA and HSPCB). | | 3 | | -14.2 | | 6.4 | 62.5 | | 5.72E+05 | | 1.83E+05 | | | |
| **3190** | | 2.5 | | 8.6 | | 40,202 | Peroxiredoxin-1 | Involved in redox regulation of the cell. Reduces peroxides with reducing equivalents provided through the thioredoxin system but not from glutaredoxin. May play an important role in eliminating peroxides generated during metabolism. Might participate in the signaling cascades of growth factors and tumor necrosis factor-alpha by regulating the intracellular concentrations of H2O2. Reduces an intramolecular disulfide bond in GDPD5 that gates the ability to GDPD5 to drive postmitotic motor neuron differentiation | | 3 | | -16.8 | | 8.3 | 22.1 | | 3.52E+06 | | 1.42E+06 | | | |
| **3255** | | 1.5 | | 8.77 | | 38,762 | Superoxide dismutase | Destroys superoxide anion radicals which are normally produced within the cells and which are toxic to biological systems. | | 5 | | -30 | | 9 | 24.7 | | 6.78E+06 | | 9.84E+06 | | | |
| **Spot No.** | **Anova (p)** | | **Fold** | | **Experimental Ip/MW (MW in KiloDaltons)** | | **Protein Description** | **Function** | **rI** | | **log(e)** | | **Theoritical Ip/MW**  **(MW in KiloDaltons)** | | | **Average Normalised Volumes** | | | | | | |
|  |  |  |  |  |  |  |  |  |  |  |  |  |  |  |  | **Rodent diet** | | **MON810** | | **Reference 1 (Mon Garst)** | **Control** | **Reference 2 (Mon Gold)** |
| **1485** | 3.10E-05 | | 3.1 | | 6.91/72.759 | | Stress-induced-phosphoprotein 1 | Mediates the association of the molecular chaperones HSC70 and HSP90 (HSPCA and HSPCB). | 3 | | -14.2 | | 6.4/62.5 | | | 3.32E+05 | | 5.72E+05 | | 1.83E+05 | 2.35E+05 | 2.40E+05 |

log(e): the base-10 log of the expectation that any particular protein assignment was made at random (E-value).

rl: the number of unique peptide sequences associated with this protein assignment.

pI: isoelectric point for the intact gene product.
